# A Novel missense mutation of *COL2A1* gene in a large family with stickler syndrome type I

**DOI:** 10.1111/jcmm.17187

**Published:** 2022-01-21

**Authors:** Xiuzhen Liu, Hongliang Dong, Yuerong Gong, Lianqing Wang, Ruyi Zhang, Tihua Zheng, Yuxi Zheng, Shuang Shen, Chelsea Zheng, Mingming Tian, Naiguo Liu, Xiaolin Zhang, Qing Yin Zheng

**Affiliations:** ^1^ Medical Research Center Binzhou Medical University Hospital Binzhou China; ^2^ Department of Ophthalmology Binzhou Medical University Hospital Binzhou China; ^3^ Center of Translational Medicine Central Hospital of Zibo Zibo China; ^4^ Department of Anesthesiology Binzhou Medical University Hospital Binzhou China; ^5^ Hearing and Speech Rehabilitation Institute College of Special Education Binzhou Medical University Yantai China; ^6^ Department of Ophthalmology Duke University Durham North Carolina USA; ^7^ Department of Otolaryngology‐HNS Case Western Reserve University Cleveland USA; ^8^ Department of Otolaryngology/Head and Neck Surgery Institute of Otolaryngology Binzhou Medical University Hospital Binzhou China

**Keywords:** *COL2A1*, ES, exon2, gender difference, nonsyndromic ocular STL1, phenotype variability

## Abstract

Stickler syndrome type I (STL1, MIM 108300) is characterized by ocular, auditory, skeletal and orofacial manifestations. Nonsyndromic ocular STL1 (MIM 609508) characterized by predominantly ocular features is a subgroup of STL1, and it is inherited in an autosomal dominant manner. In this study, a novel variant c.T100>C (p.Cys34Arg) in *COL2A1* related to a large nonsyndromic ocular STL1 family was identified through Exome sequencing (ES). Bioinformatics analysis indicated that the variant site was highly conserved and the pathogenic mechanism of this variant may involve in affected structure of chordin‐like cysteine‐rich (CR) repeats of ColIIA. Minigene assay indicated that this variant did not change alternative splicing of exon2 of *COL2A1*. Moreover, the nonsyndromic ocular STL1 family with 16 affected members showed phenotype variability and certain male gender trend. None of the family members had hearing loss. Our findings would expand the knowledge of the *COL2A1* mutation spectrum, and phenotype variability associated with nonsyndromic ocular STL1. Search for genetic modifiers and related molecular pathways leading to the phenotype variation warrants further studies.

## 1 INTRODUCTION

Stickler syndrome is characterized by ocular, auditory, skeletal and orofacial manifestations.^1^ It has been divided into three types: STL1, STL2 (MIM 604841) and STL3 (MIM 184840) with STL1 being the most common form. STL1 is caused by *COL2A1* variants and is inherited in an autosomal dominant manner. Except the familiar symptoms in eye such as high myopia, vitreoretinal degeneration, retinal detachment (RD) and cataracts, in some cases STL1 also showed short stature, scoliosis/kyphosis, joint hypermobility /osteoarthritis, cleft palate, midfacial hypoplasia and some degree of deafness.^2^, ^3^ Nonsyndromic ocular STL1 is a subgroup of STL1 and is characterized by predominantly ocular features with absent or minimal extraocular abnormalities.^4^ However, both STL1 and nonsyndromic ocular STL1 have a wide phenotypic spectrum, with considerable interfamilial and intrafamilial variability in its clinical expression.^5^

*COL2A1* locates in chromosome 12 and encodes the a1 chain of collagen II (ColIIA), which is the main component of the cartilage extracellular matrix and the vitreous of eye.^6^ The pathogenic variants of *COL2A1* cause many diseases related to dysosteogenesis, including STL1.^7^, ^8^ These variants happen in both introns and exons. While the former usually affects splice sites, the latter is much more complicated. Pathogenic variants in exons mainly contain premature stop (ie.,nonsense, frameshift) variants and missense variants. These two types of pathogenic variants cause diseases usually because of haploinsufficiency or structure defect of ColIIA, but they sometimes affect cis‐elements and alter pre‐mRNA splicing process.

In the nucleus, pre‐mRNA of pro‐ColIIA undergoes alternative splicing at exon2 and produces various isoforms: IIA, a long form that includes exon 2; IIB, a short form that excludes exon 2; IIC, which contains only the first 34 nucleotides of exon 2 and has no protein product; and IID, which contains the same sequence as IIA with an additional three nucleotides. Compared to IIA and IIB, the prevalence of IIC and IID is low, so IIA and IIB are the main isoforms.^9^ The alternative splicing event of *COL2A1* is developmentally regulated. Inappropriate splicing often causes chondrogenesis and ocular diseases. Several pathogenic variants of exon 2 related to STL1 have been found.^10^, ^11^, ^12^

The present study reports a novel pathogenic variant of *COL2A1* resulting in nonsyndromic ocular STL1 and analyses the clinical heterogeneity of this family, which enriches our understanding of nonsyndromic ocular STL1.

## 2 MATERIALS AND METHODS

### 2.1 Patient recruitment

Patients and histories were identified in Binzhou Medical University Hospital. The studies were performed with approval of the Ethics Committee of the Binzhou Medical University Hospital (2018–008–01). All persons gave informed consent prior to their inclusion in the study. Venous whole blood samples were collected for molecular genetic testing. IV‐2 and IV‐4 were missing blood samples and inspection because they were lost to follow‐up.

### 2.2 Clinical examination

Ophthalmic, orofacial, skeletal and auditory features were assessed using previously reported methods.^4^, ^13^ A general ophthalmic history was recorded, with particular attention to the age of onset, degree and progression of myopia, cataract and vitreoretinal disease. A full ophthalmic examination was performed, including slit‐lamp biomicroscopy and scleral depression with indirect ophthalmoscopy. Anterior segment photographs were taken when appropriate. Joint hypermobility was assessed objectively by the reported means of a series of tests.^14^

### 2.3 Exome sequencing (ES) and bioinformatics analysis

Genomic DNA was extracted from the peripheral leukocytes of all recruited family members using standard protocols for whole blood DNA extraction. The main portion of the ES was provided by Novogene Bioinformatics Institute. The exomes were captured using Agilent SureSelect Human All Exon V6 kits, and high‐throughput sequencing was performed in an Illumina HiSeq X‐10. The basic bioinformatics analyses, including reads, mapping, variant detection, filtering and annotation, were also provided by Novogene Bioinformatics Institute. The average coverage for all of the experiments was 70x and was at least 20x for 90% of the targets. Paired sequencing reads were aligned to the reference genome (GRCh37/hg19) using BWA 26 and sorted with SAMtools27 and Picard (http://broadinstitute.github.io/picard/webcite). Post‐alignment processing (local realignment around insertions‐deletions and base recalibration), single nucleotide variant (SNV) and small nucleotide sequences insertion‐deletion (InDel) calling were performed with Genome Analysis Toolkit (GATK) 28, with parameters adapted to the haloplex‐generated sequences. The called SNV and InDel variants produced with both platforms were annotated according to the ANNOVAR Web server (in the public domain, http://wannovar.wglab.org/index.php).^15^

A tiered filtering strategy was used to prioritize the SNVs and InDels using previously reported methods.^16^ The obtained SNVs and InDels were further analysed for conservative and possible deleterious impact by software (dbNSFP version3.0). According to a simple model of the dominant mode of inheritance pattern, we explored the SNVs that were heterozygous for the variant allele in the affected patients but normal in unaffected patients. Then, gene‐disease phenotypic correlation analysis for the candidate variants was performed, and the variants were sorted by relevance. Whether the variants involved in the splicing process were predicated in Spidex databases.

### 2.4 Pathogenic variant validation and co‐segregation analysis

Filtered pathogenic variants and co‐segregation analysis among all family members were validated by Sanger sequencing. The primer pairs were designed by Primer 5 (Table S1), and sequences of the PCR products were determined using the Eppendorf Mastercycler Genetic Analyzer. Sanger sequencing was performed at Majorbio.

### 2.5 Conservation analysis of variants in *COL2A1*

Conservation of mutant cite in *COL2A1* orthologs was analysed on UCSC (http://genome.ucsc.edu). Multiple sequence alignment analysis of CR repeats in ColIIA and other proteins was performed using DNAMAN software.

### 2.6 Construction and identification of minigenes

The minigene ‘pcDNA3.1‐WT’ was constructed containing exons 1–3 and full‐length intron 1 and intron 2 sequences of *COL2A1* according to the reported methods.^12^ Plasmid pcDNA3.1‐WT was constructed with wild‐type exon2. The point mutation (c.T100>C:p. Cys34 Arg or c.G170>A:p. Cys57Tyr) was introduced into exon 2 of pcDNA3.1‐WT using the HieffMut^TM^ Site‐Directed Mutagenesis Kit (Stratagene), to construct plasmid pcDNA3.1‐C34R or pcDNA3.1‐C57Y respectively (Figure 4A). The three recombinant plasmids were sequenced to confirm the normal and pathogenic variant clones (Figure S3).

### 2.7 Cell culture and transfection

HEK‐293 cells were cultured in DMEM containing 10% foetal bovine serum. These cells were transfected with pcDNA3.1‐WT, pcDNA3.1‐C34R and pcDNA3.1‐C57Y plasmids using Lip2000 and incubated at 37°C for 2 days before harvesting for RNA experiments.

### 2.8 Analysis of spliced mRNA isoforms derived from the *COL2A1* minigenes

Total RNA was extracted from the cells using Trizol (Invitrogen) according to the manufacturer's instructions. Total RNA was used to synthesize cDNA with a PrimeScript RT reagent kit (Takara, RR037A). Alternative splicing of the minigenes produced either the IIA or IIB isoform. The primer pairs RT‐F and RT‐R (Table S1) were used to detect the mRNA expression of IIA and IIB isoforms derived from the minigenes by RT‐PCR (Figure 4B). The products of RT‐PCR were detected by electrophoresis in 12% polyacrylamide gels and were semiquantitative analysed by ImageJ software.

### 2.9 Statistical analysis

The data were expressed as the mean ± SD of at least three independent experiments. Statistical analysis of the data was performed using one‐way ANOVA followed by Bonferroni's multiple‐comparison correction in GraphPad Prism 5.01.

## 3 RESULTS

### 3.1 Clinical presentations of the patients

**FIGURE 1:**
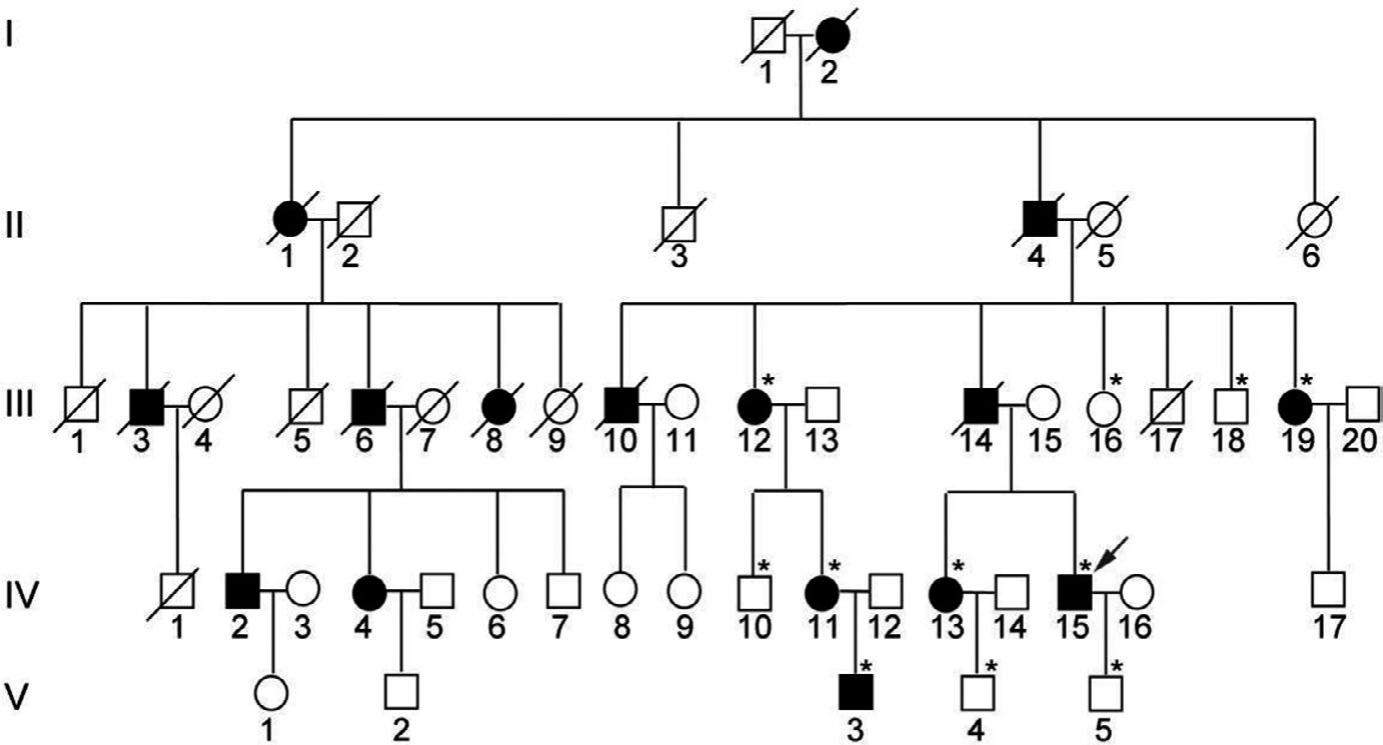
Pedigree of the nonsyndromic ocular STL1 family. The propositus was indicated by the black arrow. Normal descendants of the normal individual were not shown due to limited space. Asterisks: individuals which had blood available

**FIGURE 2:**
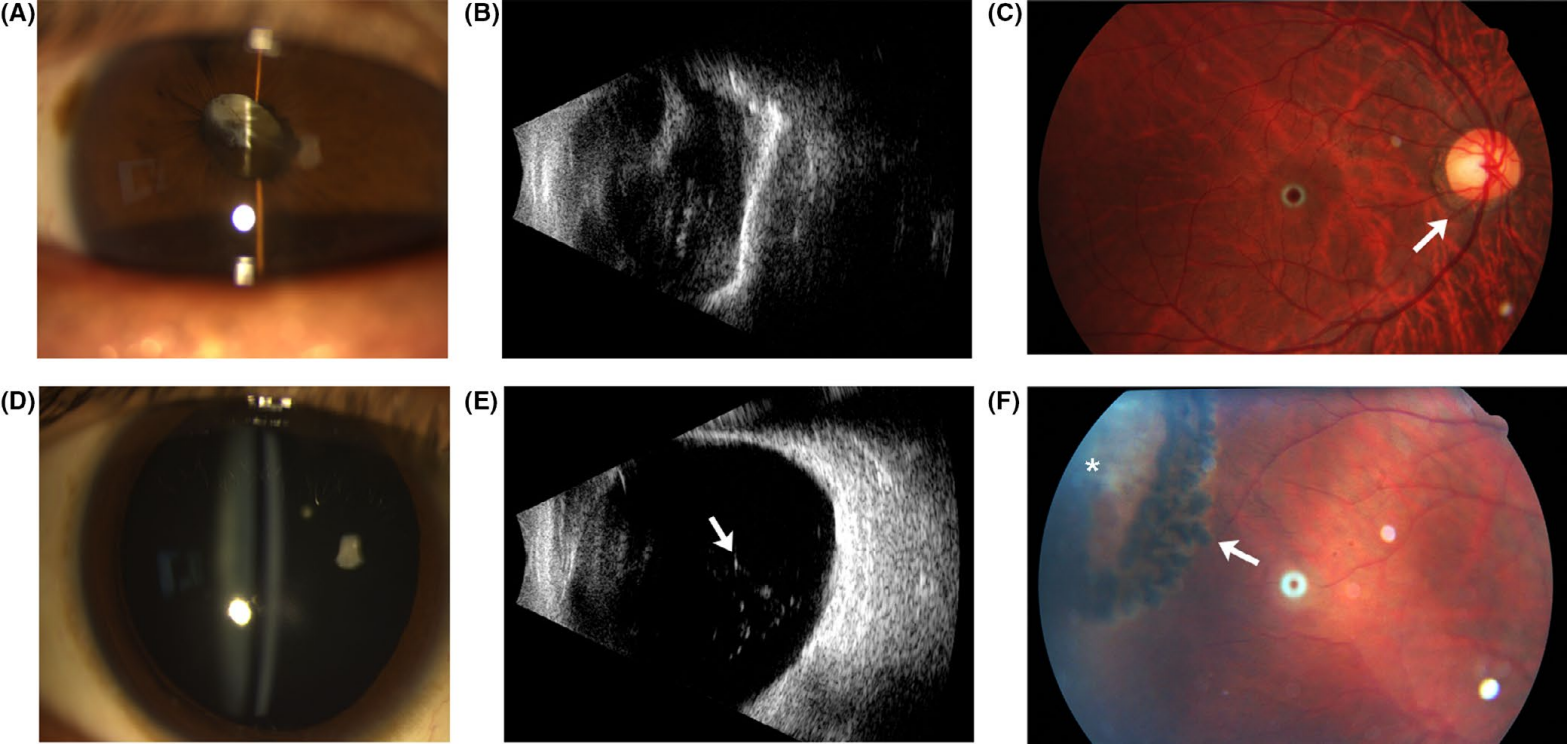
Ophthalmologic abnormalities of affected individuals of the nonsyndromic ocular STL1 family. (A, D) Anterior segment examination of IV‐15(A) and V‐3(D). (B, E)Vitreous ultrasound inspection of IV‐15(B) and V‐3(E). White arrow: dot opacification. (C, F) Fundal examination of V‐3. White arrow in (C): myopia arc. White arrow in (F): laser spots. Asterisk: retinal tear. Because IV‐15 was blind due to a retinal detachment, the segment examination was performed without pupil dilation and fundal examination was not performed

The family reported in the present study was from Shandong Province of China, and it was a five‐generation nonsyndromic ocular STL1 family with 16 affected and 33 unaffected members (Figure 1). Individual IV‐15 (the propositus) had extreme myopia in both eyes at about age 6. At age 9, this patient suffered a giant‐tear retinal detachment in the left eye that was not successfully repaired. Two years later, this patient developed a cataract in this eye. At age 10, he suffered a giant‐tear retinal detachment in the right eye and underwent retinal repair surgery. At age 11, a complicated cataract appeared in the right eye, and the retina detached, leaving faint light perception. At age 36, the left eye was enucleated due to inflammation. Now, at age 45, a slit‐lamp biomicroscopy examination for anterior segment of the right eye showed that the oval pupil moved up to temporal, and the residual lens cortex and capsule were clouded and subluxated in the anterior vitreous (Figure 2A). Ultrasound inspection showed vitreous opacities and long‐standing retinal detachment (Figure 2B). On joint examination, this patient could easily touch his fingers to the floor with extended knees despite a herniated disc (Figure S1A,B). When he was younger, he was able to easily maintain his palms to the floor. Furthermore, on examination he was able to oppose his thumb to the ventral aspect of his forearm, while the same age control could not do this (Figure S1C,D). Given this, individual IV‐15 met the diagnostic criteria of joint laxity.^14^

Individual IV‐11 had moderate myopia in the right eye at about 6 years old. Now, at age 48, the optic examination shows that: for eyesight: OD (right eye): 0.2 (−2.00–0.75 × 557° =0.4), OS (left eye): 0.6 (−0.75 × 105° =1.0); for axial length (also named anteroposterior axis), OD: 22.76 mm (millimeter), OS: 22.02 mm. Slit‐lamp examination for the anterior segment showed normal anterior‐chamber drainage‐angle development and membranous vitreous (Figure S2A). Supersonic inspection showed mild dot opacification in vitreous humor (Figure S2B). Fundus examination showed myopia characteristics of leopard fundal and myopia arc around pupil (Figure S2C). Her joint examination was normal.

Individual V‐3, the son of individual IV‐11, exhibited extreme myopia in both eyes at 3 years old. At age 13, he suffered retinal tearing in both eyes, which were repaired with laser treatment. Now, at age 23, the optic examination shows that, for eyesight, OD: (−11.75−0.75 × 175° =0.6), OS: (−8.50−1.50 × 40° =0.6); for axial length, OD: 27.48 mm, OS: 26.26 mm; and for lens thickness, OD: 3.91 mm, OS: 3.88 mm. Slit‐lamp examination for the anterior segment showed normal anterior‐chamber drainage‐angle development, clear crystalline lenses and normal membranous vitreous (Figure 2D). Ultrasound inspection demonstrated obvious dot opacification in vitreous humor, irregular circles of vitreous body and posterior staphyloma, which were high myopia characteristics (Figure 2E). Fundus examination showed that retina was attached, and characteristics of myopia such as leopard fundal and myopia arc around pupil (Figure 2C). Examination revealed retinal tears surrounded by laser spots in the peripheral retina (Figure 2F). Her joint examination was normal.

The other affected members of this family showed various degrees of myopia and retinal detachment (Table 1). All of the patients had normal stature, hearing, speech development and intelligence, and did not have cleft palate or midfacial hypoplasia.

**TABLE 1:** Evaluation of phenotypic severity of different affected individuals

Patient	Gender			Retinal detachment				Joints activity
		Myopia		Left		Right		
		Left	Right	Age(y)	Grade	Age(y)	Grade	
PI−2	F	2	2	N	0	N	0	–
PII−1	F	3	3	>50	1	>50	1	–
PII−4	M	3	3	>50	1	N	0	–
PIII−3	M	3	3	20s	2	20s	2	
PIII−6	M	3	3	18–19	2	18–19	2	–
PIII−8	F	2	2	N	0	N	0	–
PIII−10	M	3	3	16–17	2	N	0	–
PIII−12	F	3	3	N	0	>50	1	–
PIII−14	M	3	3	15–19	2	15–19	2	–
PIII−19	F	1	3	N	0	N	0	–
PIV−2	M	3	3	12–13	3	12–13	3	–
PIV−4	F	3	3	13	3	12–13	3	–
PIV−11	F	2	2	N	0	N	0	0
PIV−13	F	3	3	13	3	21	2	0
PIV−15	M	3	3	9	3	11	3	1
PV−3	M	3	3	13^*^	3^*^	13^*^	3^*^	0

Note

Score of various phenotypes: Left: the left eye; Right: the right eye. Myopia: 0: normal eyesight; 1: mild myopia,diopter<300; 2: moderate myopia, diopter 300–600; 3: high myopia, diopter>600. Retinal detachment: Age: the age at onset of retinal detachment, y: years old; >: above some years old. N: retinal did not detach. Grade: Score of retinal detachment. 0: retinal did not detach; 1: retinal detached at age older than 50 years old; 2: retinal detached at age 15–50 years old; 3: retinal detached at before 15 years old; *: suffered retinal tearing, and retinal would detached if this patients had not be treated appropriately as his eldership. Joint activity:0: normal; 1: excessive joint movement; ‐: unknown.

### 3.2 A novel pathogenic variant of *COL2A1* was identified in the nonsyndromic ocular STL1 family

**FIGURE 3:**
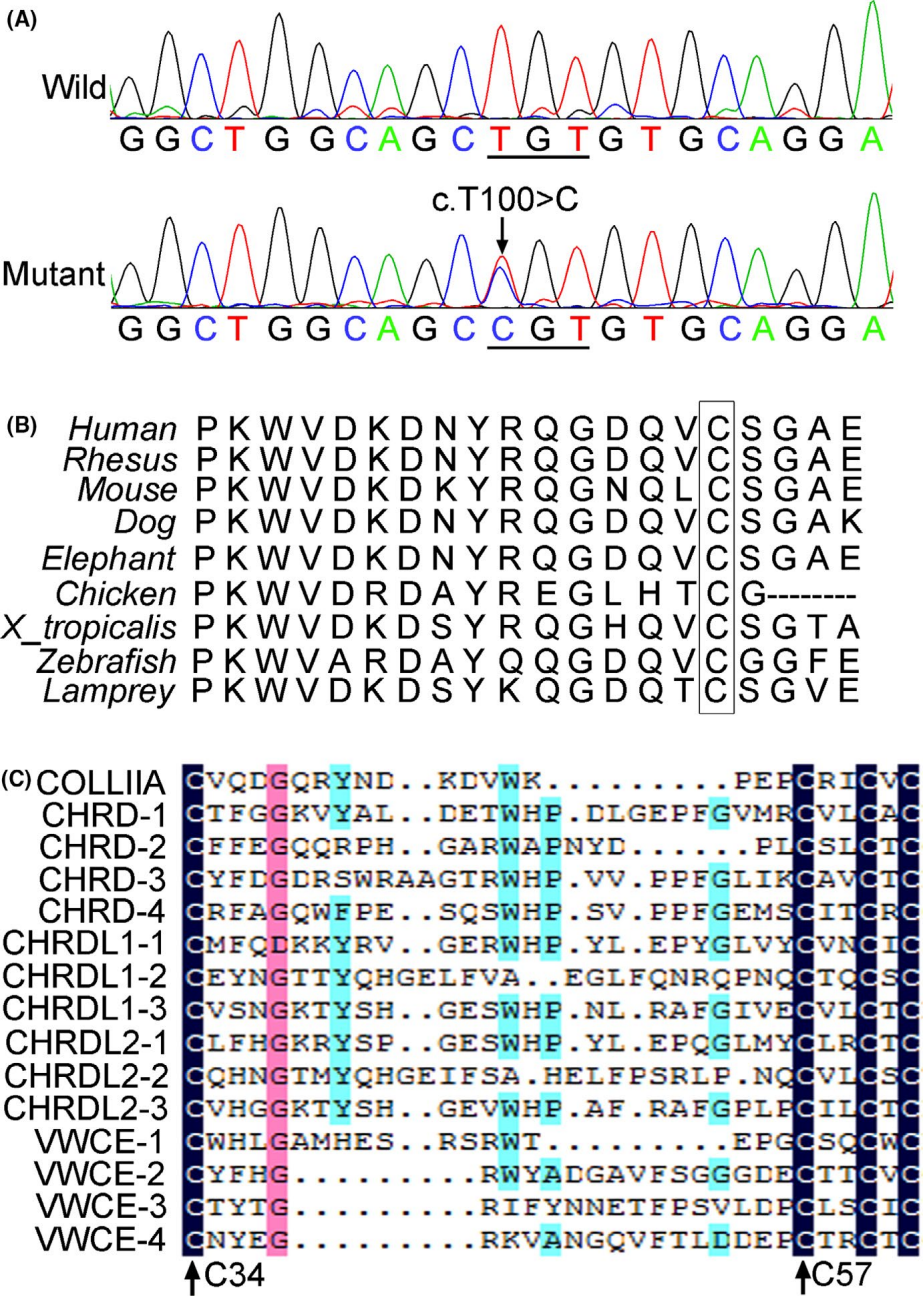
Genetic mutation identified in the nonsyndromic ocular STL1 family. (A) A heterozygous *COL2A1* variant c.T100>C (p. Cys34Arg) in exon 2 was identified in the affected members (Mutant). Arrow indicated the site of the heterozygous missense mutation. Wild indicated the wild‐type sequence in unaffected family members. (B) Multiple sequence alignment of the *COL2A1* across species.C34 was strongly conserved in ColIIA orthologs, which was displayed in the box. (C) C34 was strongly conserved in CR repeats of ColIIA, CHRD, CHRDL1, CHRDL2 and VWCE. The proteins except ColIIA have several CR repeats labeled by Arabic numbers respectively. The amino acid residues represented by small letters at the bottom row including C34 and C57 that noted by black arrow were highly conserved

ES was performed on four individuals of the pedigree, including three affected family members (III‐12, IV‐13 and V‐3) and one healthy individual (IV‐10) (Figure 1). After several prioritization steps, we obtained 21 candidate genes carrying SNVs and nine candidate genes carrying InDels, and ranked these genes according to their relevance to STL1. Then, we selected the top two of these genes which might cause STL1 directly: *COL2A1* and *COL11A2*. Sanger sequencing revealed that only the variant c.T100>C (p. Cys34Arg) in *COL2A1* was identified and co‐segregated with other family members (Figure 3A). This SNV is a novel pathogenic variant of *COL2A1*.

### 3.3 The cysteine34(C34) was highly conserved in ColIIA orthologs of various species and CR repeats of different proteins

We compared the ColIIA orthologs and found that C34 was highly conserved in various species (Figure 3B). CR repeats also known as Von Willebrand factor type C (VWC) domains^17^ are ~60–80 amino acids in length and are mainly defined by a consensus sequence of 10 cysteines, which have been identified in ~200 extracellular matrix proteins. As matrix protein, ColIIA contains CR repeat which is encoded by exon2 of *COL2A1*, and might be involved in bone and eye development through binding transcription factors by CR repeats. Therefore, variants in exon2 often are pathogenic.^18^, ^19^ We exerted multiple sequence alignment of CR repeat of ColIIA and other proteins containing CR repeats, such as CHRD, CHRDL1, CHRDL2 and VWCE. The results showed that several cysteines including C34 and C57 were strongly conserved (Figure 3C), which suggests that they are very important to maintain the structure of this domain. We also performed deleterious prediction and conservation annotation of the variant using several general international software. The results showed that the c.T100>C(p. Cys34Arg) variant of *COL2A1* changed the protein's structure or function with high probability and the mutant site was highly conserved (Table S2). These results suggested that the pathogenic mechanism of this novel variant might be involved in changes in protein structure.

### 3.4 Pathogenic variant of c.T100>C in COL2A1 did not change alternative splicing of exon 2

Exon2 of *COL2A1* pre‐mRNA undergoes alternative splicing, which produces two main isoforms: IIA and IIB.^20^ Some variants in exon 2 of *COL2A1* are often associated with Stickler syndrome, such as c.G170>A (p. Cys57Tyr) and c.G192>A (Cys64Stop), and these variants result in a shift in alternative splicing pattern towards the IIB isoform, which causes a lower ratio of IIA/IIB.^12^ While since the variant of c.T100>C (p. Cys34Arg) also located in exon2 and caused similar clinical phenotype, it might have the same mechanism to the reported pathogenic variants. To confirm the pathogenic mechanism of this variant, we predicted whether the c.T100>C (p. Cys34Arg) and c.G170>A (p. Cys57Tyr) variants in *COL2A1* involved in the splicing process using Spidex databases. In general, it is thought that the variant affects splicing when the score greater than 4 or less than −4; however, the score of the two variants could not meet this standard (Table 2), which indicated that these variants affected splicing with low probability.

**TABLE 2:** Splicing function prediction of the variants in *COL2A1*

Variants	Ref	Alt	dpsi_max_tissue
C34R	A	G	−0.2304
	A	C	−0.4974
	A	T	−0.5191
C57Y	C	T	0.6275
	C	A	−0.903
	C	G	−0.4273

Note

C34R represented a variant: c.T100>C (p. Cys34Arg), C57Y represented variant: c.G170>A (p. Cys57Tyr). Ref: base of wild type. Alt: base of all possible mutations. Dpsi_max_tissue is the score of prediction derived from Spidex databases, and it is considered that the variant affected splicing when the score greater than 4 or less than −4 generally.

**FIGURE 4:**
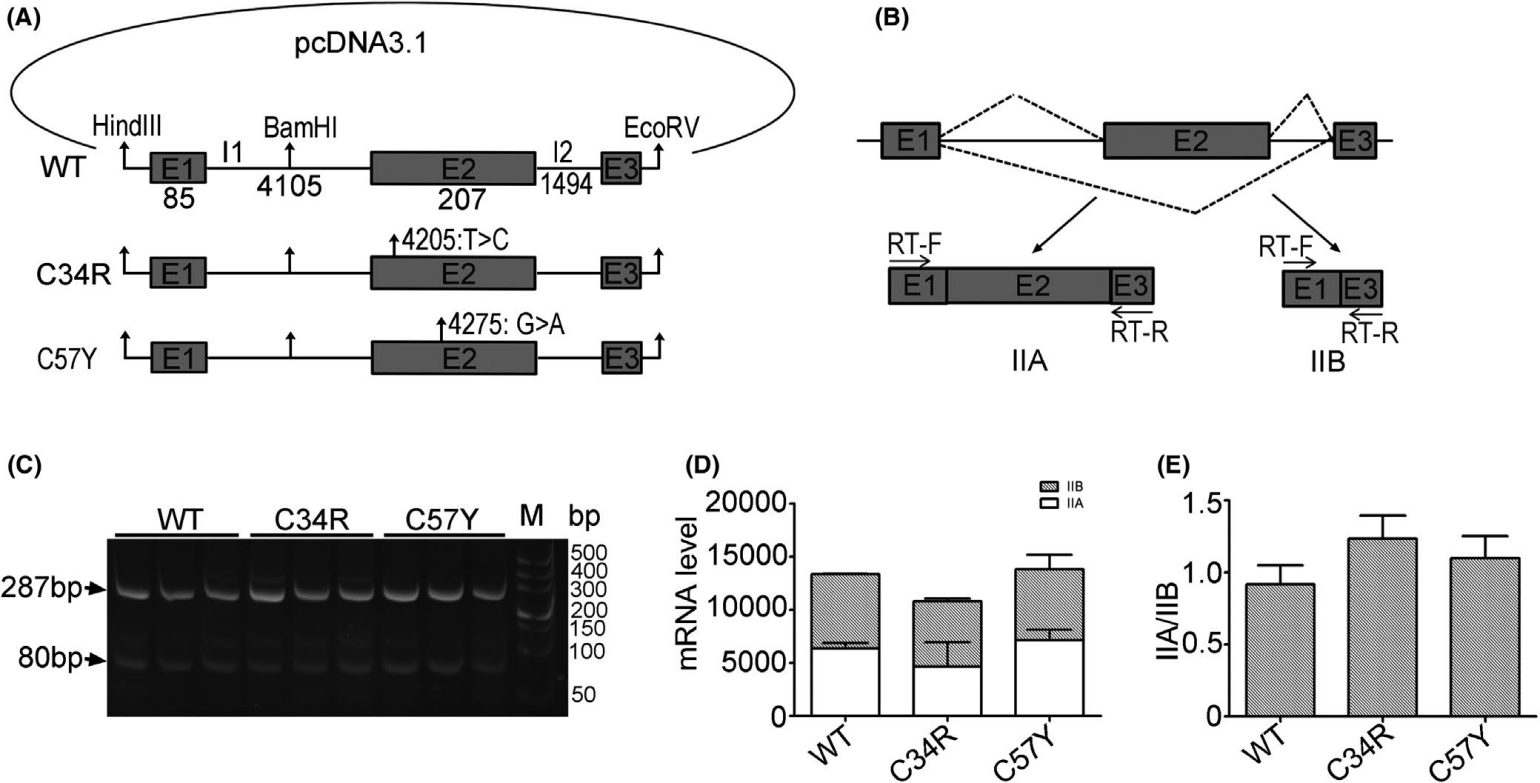
Alternative splicing of wild‐type and variants in exon 2 of the *COL2A1* E1‐E3 minigenes. (A) Construction of plasmids pcDNA3.1‐WT, pcDNA3.1‐C34R, and pcDNA3.1‐C57Y. The wild‐type minigene pcDNA3.1‐ WT contains exons 1–3 and full‐length intron 1 and intron 2 of *COL2A1*. The novel variant c.T100>C (p. Cys34Arg) was inserted into pcDNA3.1‐C34R. The confirmed pathogenic variant c.G170>A (p. Cys57Tyr) was inserted into pcDNA3.1‐C57Y. The arrows show the positions of various pathogenic variants. The numbers under exons and introns represent the nucleotide sizes of the exon/intron regions, and numbers beside the arrows above exon2 represent the variant location in *COL2A1*. (B) Mechanisms of alternative splicing of the minigene to produce either the IIA or IIB isoform. The primers RT‐F and RT‐R were used to amplify the cDNA of the two isoforms produced by the minigenes. The fine arrows show the positions of the primers. (C) RT‐PCR products of IIA and IIB spliced isoforms derived from the wild‐type and mutant minigenes. Arrow: 256bp indicated IIA, 50bp indicated IIB. WT: pcDNA3.1‐WT; C34R: pcDNA3.1‐ C34R; C57Y: pcDNA3.1‐C57Y. (D, E) Semiquantitative analysis of the RT‐PCR results of (C): (D)the relative mRNA expression levels of IIA and IIB isoforms, (E) The ratio of mRNA levels of IIA/IIB (*n* = 3)

Furthermore, we constructed *COL2A1* minigenes, and they represented wild type, c.T100>C (p. Cys34Arg) and c.G170>A (p. Cys57Tyr) variants respectively (Figure 4A, Figure S3). Then, the three minigenes were transfected into HEK‐293 cells, and the transcription products were analysed by RT‐PCR. The results showed that the three minigenes all expressed two kinds of mRNA: IIA and IIB (Figure 4B). The expected sizes of the spliced isoforms IIA and IIB derived from RT‐PCR were shown in (Figure 4C), and expression level of isoforms IIA was higher than isoforms IIB in all minigenes. The total expression quantities of IIA and IIB and the ratios of IIA/IIB of the three minigenes showed no difference (Figure 4D,E). These results suggested that the c.T100>C (p. Cys34Arg) and c.G170>A (p. Cys57Tyr) variants in *COL2A1* did not alter the alternative splicing of exon2, which was different from the previous reports.

### 3.5 Phenotypes of this nonsyndromic ocular STL1 family showed significant heterogeneity and more serious in male gender

STL1 has obvious heterogeneity in clinical manifestations.^5^ In our study, the nonsyndromic ocular STL1 family showed a wide phenotypic spectrum (Table 1). The clinical manifestations were mainly in eye and joint, and the abnormalities in eye were myopia and retinal detachment mainly, while the joint abnormality was joints flabby indicated by excessive joint movement. In this family, all of the affected members showed myopia, but the severity varied widely from mild myopia (diopter<300) to high myopia (diopter>600). Manifestations in the retina were different from not detached to detached at various ages which indicated variance in severity. Most of the affected members’ joints were normal, and a few were unknown because they were very old or had died, with most severely lax joints in IV‐15.

We graded the clinical manifestations according to its severity, as shown in Table 1, the most mildly affected members who had the lowest grades were I‐2, III‐8, III‐19 and IV‐11, and they showed only mild myopia in general. The most severely affected members who had high grades were IV‐2, IV‐4, IV‐15 and V‐3, and they showed extreme myopia, retinal detachment in both eyes before 15 years old and the joints were lax occasionally (Figure 2, Table 1, Figures S1,S2).

**FIGURE 5:**
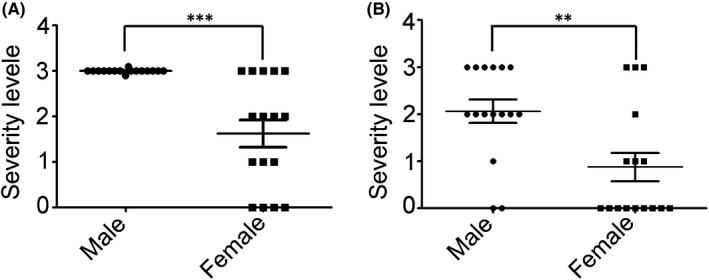
Clinical phenotypes showed more serious in male than that in female in both eyes. (A) Compare the serious degree of myopia between male and female in the left and right eyes. (B) Compare of serious degree of retinal detachment between male and female in left and right eyes

We then analysed the heterogeneity in clinical manifestations in different genders of this nonsyndromic ocular STL1 family. The results showed that all of the affected male members of the STL1 family had high myopia in both eyes; however, a few female members only showed mild myopia (Figure 5A). Furthermore, the severity of retinal detachment between male and female patients in the left and right eyes showed a similar trend. Males had more retinal detachments and had them earlier in life than females (Figure 5B).

## 4 DISCUSSION

In this report, we identify a novel pathogenic variant (c.T100>C, p. Cys34Arg) in *COL2A1* in a nonsyndromic ocular STL1 family. The cysteine at amino acid 34 was highly conserved in ColIIA orthologs of various species and in CR repeats of various proteins, and the variant did not involve in the splicing process. We also analysed the clinical heterogeneity of this family and found that the phenotypic severity showed a certain gender trend.

Pre‐mRNA of *COL2A1* is alternatively spliced during development, and IIA isoforms express predominantly in embryonic cartilage, while IIB isoforms express mainly in adult cartilage.^21^, ^22^, ^23^ This switch between the IIA and IIB splice forms also have been identified in the vitreous, and it is reported that the ratio of IIA/IIB was 5:1 in fetal vitreous, while it became 1.5:1 in adult vitreous of the bovine eye.^18^, ^24^ Several pathogenic variants of *COL2A1* associated with STL1 made a decreased ratio of isoforms IIA/IIB.^12^ However,in our study, the minigene assay showed that the c.T100>C (p. Cys34Arg) variant of *COL2A1* did not alter the expression level of IIA and IIB and their ratio. Interestingly, the variantc.G170>A (p. Cys57Tyr) caused STL1 through a decreasing ratio of isoforms IIA/IIB in the previous report; however, the ratio did not change in our study. Furthermore, bioinformatics analysis which is commonly accepted methods also supported our results, so we believe that both the novel variant c.T100>C (p. Cys34Arg) and the reported variant p. Cys57Tyr causes STL1 not through affecting alternative splicing, which is different from the previous reports.

The extracellular matrix provides a structural scaffold that imparts physical properties to connective tissue, and they also act as an instructive platform for soluble modulators of cell behaviour.^25^ TGF‐β (transforming growth factor‐beta) binds to extracellular matrix macromolecules and plays an important role in embryonic tissues, and its dysregulation involved in the pathogenesis of retinal detachment.^26^ BMP4 (bone morphogenetic protein 4) is a secreted ligand of the TGF‐β superfamily and plays critical role in ocular development.^27^, ^28^ In recent report, a novel heterozygous BMP4 variant was first identified to cause STL1.^29^ The CR repeats can bind members of TGF‐β superfamily and are proposed to regulate growth factor signalling. ColIIA contains a 69‐amino acid chordin‐like CR repeat, which encoded by exon2 that exists in type IIA exclusively.^22^ ColIIA interacts with proteins of the TGF‐β superfamily by CR repeat and therefore potentially regulates growth factor signalling during development.^18^ The isolated CR ColIIA repeat binds BMP‐2 and TGF‐β in solid‐phase binding assays,^19^ and binding to BMP‐4 is competed by BMP‐2 and TGF‐β.^30^ So the CR repeat of ColIIA plays a role during development through regulating growth factor signalling, and changes in its sequence or structure may cause abnormal development of related organs or tissues. Pathogenic variants in exon 2 of the *COL2A1* gene often cause nonsyndromic ocular STL1, such as the pathogenic variants Gly67Asp, Trp47Ter, Cys64Ter and Cys57Tyr.^10^, ^11^, ^12^ Additionally, intronic sequence variants affecting the alternative splicing efficiency of exon 2 of *COL2A1* have been associated with an increased risk of retinal detachment.^31^ In this study, we found C34 amino acid was highly conserved in different species and in multiple CR repeats of different proteins. O’Leary et al. speculate the solution structure and dynamics of a prototypical CR repeat from ColIIA, and found Cys34 and Cys57 formed disulphide bond, which is important to maintain the structure of the CR repeat.^32^ So, all of the analysis suggests that the variant c.T100>C (p. Cys34Arg) mutation of *COL2A1* might dysregulate the growth factors through altering the structure of the CR repeat.

While gender trends for clinical manifestations of SLT1 have been rarely described in literature thus far, there have been a few reports describing gender trends for both myopia and RD. Several studies suggest a higher prevalence of nonpathological myopia among females compared to males.^33^, ^34^ However, in Hyman's report, myopia progressed in females only slightly more than in males.^35^ Contrarily, Chen found that testosterone levels positively correlate with high myopia both in males and females; progesterone was negative correlation in contrast. Additionally, he reported that levels of estradiol were significantly higher in myopic males, but lower in myopic females.^36^ However, despite these studies demonstrating female trend in myopia, several studies have reported that male sex is associated significantly with greater risk of RD.^37^, ^38^, ^39^ Incidence of RD after cataract surgery or Pars Plana Vitrectomy also appeared increased in male gender.^40^, ^41^, ^42^ Male sex was reported as a risk factor for pseudophakic retinal detachment after cataract extraction in Taiwanese adults.^43^ However, one report demonstrated a contrary results that significant risk factors for recurrent retinal detachment include size of retinal tear, age, prior vitrectomy and female gender.^44^ From the above reports, it is difficult to definite the relationship between RD and gender, but several studies suggest that RD might have a higher incidence in males. We report that many major characters of STL1 including myopia and RD seem to have an association with gender. In our study, the affected male members displayed more serious myopia than females, which is different from the trend of nonpathological myopia. Perhaps with this particular genetic background, the progression of myopia is more sensitive to the sex hormones such as testosterone, which was found at a higher level in males. Furthermore, RD in males was more serious than in female in our study, possibly due to more serious high myopia, previously published male trend in RD, genes modification related to sex that have not been yet described in literature. It was reported that 32 STL1 patients complained of hearing loss (37%, 95% CI 27–48) of whom 17 required hearing aids.^45^ In contrast, our current report did not have any patients complaining of hearing loss, neither required hearing aids. Our previous report shows that a mouse model with a missense mutation in the mouse *Col2a1* gene resulted in a mouse phenotype similar to human STL1, including hearing impairment ranged from 35 to 50 dB hearing loss to completely deaf in a mixed genetic background.^8^ All of these reports suggest that genetic background plays important roles in the phenotypic variations. Search for genetic modifiers and related molecular pathways leading to the phenotype variation warrants further study.

In summary, in this study, we identified a novel missense pathogenic variant in *COL2A1* in a nonsyndromic ocular STL1 family. The pathogenic mechanism of this variant may be involved in protein structure changing. The nonsyndromic ocular STL1 family showed clinical variability and a male gender trend with unknown reason. Our findings expand the knowledge of the *COL2A1* mutational spectrum and clinical heterogeneity associated with STL1.

## CONFLICT OF INTEREST

The authors declare that there is no conflict of interests to report.

## AUTHOR CONTRIBUTIONS

**Xiuzhen Liu:** Conceptualization (equal); Investigation (equal); Project administration (equal); Writing – original draft (equal). **Hongliang Dong:** Investigation (equal); Methodology (equal); Project administration (equal); Writing – original draft (equal). **Yuerong Gong:** Investigation (equal); Methodology (equal). **Lianqing Wang:** Formal analysis (equal); Methodology (equal); Supervision (equal). **Ruyi Zhang:** Data curation (equal); Resources (equal). **Tihua Zheng:** Funding acquisition (equal); Investigation (equal); Validation (equal). **Yuxi Zheng:** Data curation (equal); Supervision (equal). **Shuang Shen:** Formal analysis (equal); Software (equal). **Chelsea Zheng:** Formal analysis (equal); Supervision (equal). **Mingming Tian:** Methodology (equal); Validation (equal). **Naiguo Liu:** Data curation (equal); Formal analysis (equal). **Xiaolin Zhang:** Conceptualization (equal); Investigation (equal); Supervision (equal). **Qingyin Zheng:** Conceptualization (equal); Funding acquisition (equal); Investigation (equal); Supervision (equal).

## Supporting information

Supplementary Material

## Data Availability

All data that support the findings in this study are included in this published article and its supplementary information files.
