# Supplementary material for: A Novel missense mutation of COL2A1 gene in a large family with stickler syndrome type I
Source: J Cell Mol Med. 2022 Jan 21;26(5):1530–9. doi: 10.1111/jcmm.17187 (PMC8899160; doi:10.1111/jcmm.17187)
Supplement: Supplementary file 1 — Supplementary Material [file JCMM-26-1530-s001.docx]

**Supplement figures and tables:**





FIGURE S1 Articular movement examination of individual IV-15 of the nonsyndromic ocular STL1 family and the normal control. (A, B) Forward flexion of the trunk, with knees straight, the hands tried to touch the floor. A: normal; B: IV-15. (C, D) Passive apposition of the thumbs to the flexor aspects of the forearms. C: normal; D: IV-15


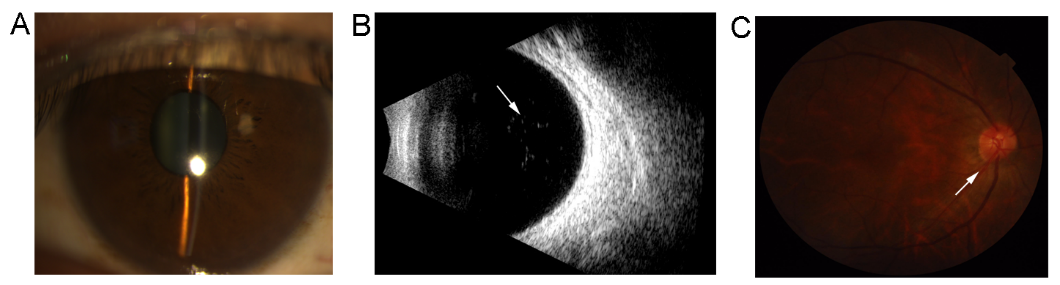


FIGURE S2 Ophthalmologic examination of individual IV-11 of the nonsyndromic ocular STL1family. (A) Anterior segment examination. (B) Vitreous ultrasound [inspection](javascript:;). White arrow: dot opacification. (C)Fundal examination. White arrow: myopia arc. IV-11 only had moderate myopia, so their anterior segment examination performed without pupil dilation.

**
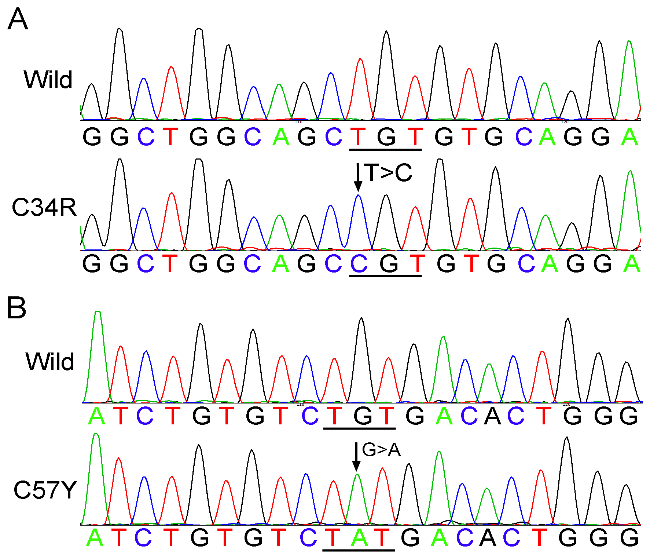
**

FIGURE S3 Sequence of the mutant minigenes. (A) (pcDNA3.1-C34R): the novel pathogenic variant Cys34 Arg (T>C). (B) pcDNA3.1-C57R: the reported pathogenic variant Cys57Tyr (G>A). Bases with Gray background are the mutant base.

TABLE S1 Primers used for mutation analysis and RT-PCR

| Human Genes | Primer pairs（5’- 3’） | | Product size |
| --- | --- | --- | --- |
| *COL2A1*-exon2 | F | CCCCAGCCTACATTCTTCAGC | 480bp |
|  | R | AGTGGCCTTTCCTTTCTACCC |  |
| *COL11A2*-exon5 | F | TCCTTGTTCTGCCAACTTTCC | 595bp |
|  | R | CTCCCAGCCACAAATTCTTCA |  |
| *COL17A1*-exon4 | F | CAGCAGCGGCTACATAAACTCAA | 931bp |
|  | R | AGTACACTCAGGGAGGGTCTTCG |  |
| *COL17A1*-exon5 | F | GGAGGTCCAGAAATAAGTAACAGG | 546bp |
|  | R | GAAAGCAAGCAATAGTGGAGGTG |  |
| Mini-gene-RT | F | AGACGCTGGTGCTGCTG | 287bp; 80bp |
|  | R | CTTTGGTCCTGGTTGCC |  |

F:[forward](javascript:;) [primer](javascript:;); R:reverse primer

TABLE S2 ANNOVAR [annotation](javascript:;) of the c.T100>C (p.Cys34Arg) variant of *COL2A1*

|  | [Annotation](javascript:;) way | Score | Result of prediction |
| --- | --- | --- | --- |
| 1 | SIFT | 0.002 | D |
| 2 | Polyphen2_HVAR | 0.99 | D |
| 3 | Polyphen2_HDIV | 0.991 | D |
| 4 | MutationTaster | 1 | D |
| 5 | LRT | 0.000 | D |
| 6 | MutationAssessor | 4.295 | H |
| 7 | FATHMM | -5.81 | D |
| 8 | SiPhy_29way_logOdds | 13.325 | H |
| 9 | phyloP46way_placental | 2.09 | H |
| 10 | phyloP100way_vertebrate | 7.259 | H |

1-7: Deleterious [annotation](javascript:;): Deleterious prediction of the mutation using general international software. D: Deleterious, H: High, both of them mean the mutant change the protein’s structure or function with high probability. 8-10: Conservation [annotation](javascript:;): Conservation analysis using general international software. High means the mutant site is high conservation.
